# The geographic distribution patterns of HIV-, HCV- and co-infections among drug users in a national methadone maintenance treatment program in Southwest China

**DOI:** 10.1186/1471-2334-14-134

**Published:** 2014-03-10

**Authors:** Yi-Biao Zhou, Song Liang, Qi-Xing Wang, Yu-Han Gong, Shi-Jiao Nie, Lei Nan, Ai-Hui Yang, Qiang Liao, Xiu-Xia Song, Qing-Wu Jiang

**Affiliations:** 1 Department of Epidemiology, School of Public Health, Fudan University, 138 Yi Xue Yuan Road, Shanghai 200032, China; 2 Key Laboratory of Public Health Safety, Ministry of Education (Fudan University), 138 Yi Xue Yuan Road, Shanghai 200032, China; 3 Tropical Disease Research Center, Fudan University, 138 Yi Xue Yuan Road, Shanghai 200032, China; 4 Department of Environmental and Global Health, College of Public Health and Health Professions, University of Florida, Gainesville, FL 32610, USA; 5 Emerging Pathogens Institute, University of Florida, Gainesville, FL 32610, USA; 6 Center for Disease Prevention and Control of Liangshan Prefecture, Sichuan, China

**Keywords:** HIV, HCV, Co-infection, Geographic distribution, Geographic autocorrelation analysis, Geographic scan statistic

## Abstract

**Background:**

HIV-, HCV- and HIV/HCV co-infections among drug users have become a rapidly emerging global public health problem. In order to constrain the dual epidemics of HIV/AIDS and drug use, China has adopted a methadone maintenance treatment program (MMTP) since 2004. Studies of the geographic heterogeneity of HIV and HCV infections at a local scale are sparse, which has critical implications for future MMTP implementation and health policies covering both HIV and HCV prevention among drug users in China. This study aimed to characterize geographic patterns of HIV and HCV prevalence at the township level among drug users in a Yi Autonomous Prefecture, Southwest of China.

**Methods:**

Data on demographic and clinical characteristics of all clients in the 11 MMTP clinics of the Yi Autonomous Prefecture from March 2004 to December 2012 were collected. A GIS-based geographic analysis involving geographic autocorrelation analysis and geographic scan statistics were employed to identify the geographic distribution pattern of HIV-, HCV- and co-infections among drug users.

**Results:**

A total of 6690 MMTP clients was analyzed. The prevalence of HIV-, HCV- and co-infections were 25.2%, 30.8%, and 10.9% respectively. There were significant global and local geographic autocorrelations for HIV-, HCV-, and co-infection. The Moran’s I was 0.3015, 0.3449, and 0.3155, respectively (*P* < 0.0001). Both the geographic autocorrelation analysis and the geographic scan statistical analysis showed that HIV-, HCV-, and co-infections in the prefecture exhibited significant geographic clustering at the township level. The geographic distribution pattern of each infection group was different.

**Conclusion:**

HIV-, HCV-, and co-infections among drug users in the Yi Autonomous Prefecture all exhibited substantial geographic heterogeneity at the township level. The geographic distribution patterns of the three groups were different. These findings imply that it may be necessary to inform or invent site-specific intervention strategies to better devote currently limited resource to combat these two viruses.

## Background

Human immunodeficiency virus (HIV) infection and hepatitis C virus (HCV) infection are major public health problems worldwide. More than 34 million persons currently live with HIV/AIDS and 170 million people may be infected with HCV [1-3]. By the end of 2011, China had an estimated 780,000 (620,000-940,000) people living with HIV/AIDS including 154,000 (146,000-162,000) AIDS cases. 28.4% of the 780,000 persons were infected from intravenous drug use [4]. In 2011, an estimated 48,000 individuals were newly infected with HIV, and in the same year 28,000 people died from AIDS [4]. As HIV and HCV share similar routes of transmission, including blood-blood contact, injected drug use, and sexual contact, co-infection with HIV and HCV is very common. Intravenous drug users (IDUs) often share contaminated needles or syringes for intravenous drug injection [1-3,5-7]. It has been estimated that 25% of people infected with HIV in the United States are also infected with HCV. The reported prevalence of co-infection with HIV and HCV is above 90% among IDUs [5,8,9]. The accelerated liver disease found in HCV patients leads to increased morbidity and mortality in the HIV/AIDS patients. Co-infected patients bear a significant proportion of the mortality [1].

In China, there are approximately 2.4 million IDUs, the world’s largest population of IDUs. The proportion of IDUs living with HIV/AIDS is high [10]. In 1989, the first HIV outbreak in China appeared among IDUs from Southwest China. This has become one of the most severe HIV/AIDS epidemic areas in China [4,10]. Since then, China has undergone an ever-growing increase in HIV/AIDS prevalence, initially fuelled by IDUs. In 2001, up to 66.5% of newly diagnosed HIV infections were related to drug use [11]. In order to constrain the dual epidemics of HIV/AIDS and drug use, China has adopted a methadone maintenance treatment program (MMTP) [11]. The MMTP was initiated in early 2004 as a small pilot project in just eight government-supported clinics of five provinces, and subsequently expanded into a nationwide program encompassing 738 clinics covering 27 provinces and serving some 344,254 drug users by the end of 2011 [11,12]. Numerous studies reported the HIV and HCV infection prevalence among MMTP clients in China, showing the large variations in prevalence of HIV and HCV infection in different geographic locations. However, few studies have analyzed the geographic distribution patterns of HIV and HCV infections on a smaller scale (e.g., below county level) [12]. This type of analysis is needed to better understand behavioral and risk perception factors that may contribute to infections. Advances in geographic statistical techniques and geographical information systems (GIS) provide powerful tools that help characterize and improve our understanding of the geographic distribution of diseases [13].

This study aimed to characterize the geographic distribution patterns of HIV and HCV infections among drug users at the township level using GIS-based geographic analyses involving geographic autocorrelation analysis and geographic scan statistics. The high- or low prevalence areas of HIV-, HCV- and co-infections among drug users were identified in a Yi Autonomous Prefecture, Southwest of China, where a national MMTP has been implemented since 2004. Knowledge of geographic distribution patterns of HIV and HCV infections among drug users has critical implications for future MMTP implementation and health policies regarding both HIV and HCV prevention among drug users in China. These findings will help aid the public health focus and better guide the use of limited resources.

## Methods

### Study location and population

**Figure 1:**
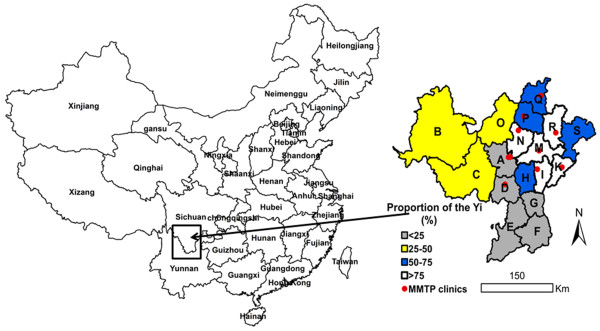
**A map illustrating the location of the Yi Autonomous Prefecture in Southwest China and positions of the 11 MMTP clinics in the Yi Autonomous Prefecture.** The box shows the position of the Yi Autonomous Prefecture. The right enlarged map shows the geographic positions of the 11 MMTP clinics and the distribution of the Yi ethnic group.

This study took place in the Yi Autonomous Prefecture, Southwest of China, one of the most endemic areas of HIV/AIDS in China. The geographic area is 6.04 × 10^6^ ha in size and has a population of 4.873 million. Approximately 50% of the population belongs to the Yi ethnic group. The Yi Autonomous Prefecture consists of 618 townships, 16 counties and 1 city. The proportion of Yi are more than 50% in 9 of 16 counties and more than 90% in the I, M and R counties (Figure 1). In 2004, one of the first eight national MMTP clinics was established in the Prefecture [14]. By the end of 2012, there were 10 fixed MMTP clinics and 1 mobile MMTP clinic. City A had two fixed MMTP clinics and 1 mobile MMTP clinic (Figure 1).

All clients attending the 11 MMTP clinics of the Yi Autonomous Prefecture from March 2004 to December 2012 were selected as our study population. Details on the eligibility for participating in the national MMTP were reported elsewhere [11,14,15]. Inclusion criteria were: 1) Heroin users failing to quit drug use more than once; 2) age ≥ 20 years; and 3) full civil capacity. All clients in MMTP clinics were checked for HIV and HCV infections one month after entry into the MMTP [14,15].

Written informed consent was obtained from all clients. Ethical approval for the MMTP was granted by the institutional review board of the National Center for AIDs/STD Control and Prevention, China CDC.

### Data collection and management

The demographic and clinical characteristics of all clients in the 11 MMTP clinics of the Yi Autonomous Prefecture from March 2004 to December 2012 were collected from the database of the national MMTP [11]. The information regarding HIV and HCV infections, gender, ethnicity, age, drug use behaviors and address of the MMTP clients were used in the study. Data were entered in Microsoft Excel version 2003 (Microsoft Corp., Redmond, WA). Individuals were considered infected with HIV or HCV if they had one positive test result when they first entered into the MMTP. Only the clients with the results of both HIV or HCV infection and a current address were analyzed.

### Statistical analysis

The characteristics and infection prevalence of the clients were analyzed using the SPSS Statistical Package for Social Sciences (SPSS Inc., Chicago, IL, 2007).

### Geographic autocorrelation analysis

Geographic autocorrelation analysis was used to detect significant differences from a random geographic distribution of HIV infection, HCV infection, and HIV/HCV co-infection. Moran’s I spatial autocorrelation statistics were calculated and visualized in the form of Moran Scatter Plots and Cluster Maps. In order to consider variance instability of prevalence in both Global and Local geographic autocorrelation analyses, the Empirical Bayes (EB) adjustment for the variance instability of infection prevalence was employed [16]. The geographic weight files were created using Contiguity-Based Geographic Weights (Rook Criterion). A significance assessment was performed by means of a permutation test, and a reference distribution was generated under the assumption that the prevalence was randomly distributed. In order to obtain more robust results, the number of permutation tests was set to 99999. Runs were carried out until the results stabilized. The significant *P*-level was set as 0.05. Both Global and Local geographic autocorrelation analyses were implemented using Open Geoda 0 · 9 · 8 · 14 software (http://geodacenter.asu.edu/software). The Local geographic autocorrelation analyses generated four geographic patterns including High-High, High-Low, Low-High and Low-Low [13] A High-High pattern was one in which a township and its surrounding townships collectively had higher infection prevalence than the average in the database. A Low-Low pattern was one in which a township and its surrounding townships collectively had below average infection prevalence. A High-Low pattern was one in which a township had above average infection prevalence, while its surrounding townships had below average infection prevalence. A Low-High pattern was one in which a township had below average infection prevalence, but its surrounding townships had above average infection prevalence.

### Linear correlation analyses

Preliminary data analyses showed that the EB adjusted prevalence of HIV-, HCV-, and co-infection was skewed, so a square root transformation of the EB adjusted prevalence was performed. The linear correlations of the HIV-, HCV- and co-infection EB adjusted prevalence across townships were analyzed and the Pearson’s correlation coefficient was calculated.

### Geographic cluster analysis

Geographic scan statistics were employed to detect and evaluate clusters with high or low prevalence of HIV, HCV and HIV/HCV cases in purely geographic dimensions. These were done by gradually scanning a circular window across geography and noting the number of observed and expected observations inside the window at each location. The Bernoulli probability model for high or low prevalence was used. The maximum geographic cluster size was set to 50% percent of the clients at risk or not at risk [17,18]. Cases were defined as the MMTP clients infected with HIV or HCV in a location. Controls were the remainder of the MMTP clients at this location. The location of cases and controls was defined as the geographical coordinate (i.e., latitude/longitude) of the center of the township in which they resided. Monte Carlo hypothesis testing [19] was employed in this study and the number of replications was set to 9999, with significance at the 0.05 level. Geographic analyses were carried out using the SaTScan™ v9 · 1 · 1 software (Kulldorff M. and Information Management Services, Inc.) (http://www.satscan.org/download.html).

A digitized polygon map was obtained for the Yi Autonomous Prefecture at a scale of 1:250,000. Clusters were mapped using ArcGIS 10.0software (Environmental Systems Research Institute, Inc., Redlands, CA) in order to identify their physical location.

## Results

### Demographic Characteristics and infection prevalence in MMTP clients

A total of 6690 MMTP clients with both home address and results of HIV or HCV testing from March 2004 through the end of 2012 were obtained from the database of the 11 MMTP clinics (Table 1). 25.2% (1633 of 6488) were HIV infected and 30.8% (1735 of 5628) were HCV infected. 10.9% (590 of 5426) were HIV/HCV co-infected. Both the HIV infection (*P* < 0.0001) and HIV/HCV co-infection (*P* = 0.004) prevalence in men were higher than that in women. There was no significance difference in HCV infection prevalence by gender. HIV (*P <* 0.0001) and HCV (*P* = 0.027) infection and HIV/HCV (*P* = 0.002) co-infection prevalence of the MMTP clients decreased with increasing age. There was a higher prevalence of both HIV infection and HIV/HCV co-infection in Yi patients than in Han patients (*P <* 0.0001). The prevalence of HCV infection in Han patients was higher than that of Yi patients (*P <* 0.0001). As the proportion of Yi patients increased at the county-level, both HIV infection (*P* < 0.0001) and HIV/HCV co-infection (*P =* 0.021) prevalence in the MMTP clients increased, while HCV infection prevalence decreased (*P <*0.0001). MMTP clients that injected drugs or shared syringes were more likely to be infected with HIV and HCV than the MMTP clients who did not inject drugs or share syringes (*P* < 0.0001) (Table 1). Multivariate logistic regression analyses showed that age was not associated with HIV infection prevalence (*χ*^*2*^_*wald*_ = 1.19*, P* = 0.276) or HIV/HCV (*χ*^*2*^_*wald*_ = 0.72, *P* = 0.396) co-infection. Patient gender was not related to HIV/HCV co-infection (*χ*^*2*^_*wald*_ = 1.04, *P* = 0.308). Other characteristics were associated with the two virus infections (*P* < 0.01).

**Table 1:** Demographic characteristics in patients with HIV infection, HCV infection and HIV/HCV co-infection at the MMTP clients in Southwest China

**Characteristics**	**HIV**		**HCV**		**HIV/HCV**	
	**Number of clients examined**	**Infection prevalence (%)**	**Number of clients examined**	**Infection prevalence (%)**	**Number of clients examined**	**Infection prevalence (%)**
Gender	*χ*^*2*^ = 36.14, *P <* 0.0001		*χ*^*2*^ = 0.78, *P* = 0.378		*χ*^*2*^ = 8.29, *P* = 0.004	
Female	928	17.2	866	32.1	825	8.0
Male	5560	26.5	4762	30.6	4601	11.4
Age (years)	*χ*_*trend*_^*2*^ = 29.77, *P <* 0.0001		*χ*_*trend*_^*2*^ = 4.87, *P* = 0.027		*χ*_*trend*_^*2*^ = 9.56, *P* = 0.002	
<30	2885	27.5	2452	31.0	2376	11.9
30-	2773	24.7	2445	32.3	2338	10.8
40-	740	20.1	654	26.2	635	8.5
≥50	90	6.7	77	18.2	77	2.6
Ethnicity	*χ*^*2*^ = 186.89, *P <* 0.0001		*χ*^*2*^ = 198.53, *P <* 0.0001		*χ*^*2*^ = 31.28, *P <* 0.0001	
Yi	5381	28.5	4492	26.5	4386	12.0
Han	1058	8.6	1089	48.2	997	5.9
Other	49	18.4	47	44.7	43	9.3
County (the proportion of the Yi (%))	*χ*_*trend*_^*2*^ = 198.30, *P <* 0.0001		*χ*_*trend*_^*2*^ = 276.11, *P <* 0.0001		*χ*_*trend*_^*2*^ = 5.37, *P =* 0.021	
<25	1072	10.7	1117	44.8	1011	7.1
25-	187	12.3	174	30.5	169	8.3
50-	1305	23.6	1244	44.0	1222	16.2
≥75	3824	30.9	2994	18.9	2925	10.2
Route of drug use	*χ*^*2*^ = 426.07, *P <* 0.0001		*χ*^*2*^ = 334.83, *P <* 0.0001		*χ*^*2*^ = 199.43, *P <* 0.0001	
Smoking or sniffing	4050	16.3	3589	22.1	3486.0	6.3
Pure injection	1114	36.5	984	48.3	910.0	18.5
Injection mixed with other	1413	40.6	1141	41.9	1115.0	18.6
Sharing syringes	*χ*^*2*^ = 435.43, *P <* 0.0001		*χ*^*2*^ = 103.30, *P <* 0.0001		*χ*^*2*^ = 162.66, *P <* 0.0001	
Yes	1176	49.1	1017	44.1	915	22.8
No	5312	19.9	4611	27.9	4511	8.4

### Characteristics of drug users of different ethnicity

More than half of all drug users smoked or sniffed drugs. The proportion of Han patients that only injected drugs was higher than that of other ethnicities (*χ*^*2*^ = 258.61, *P <* 0.0001). More than half of drug users did not share syringes. The proportion of the Han patients sharing syringes was higher than that of Yi patients (*χ*^*2*^ = 67.69 *P <* 0.0001) (Table 2).

**Table 2:** Characteristics of drug users among different ethnic groups

	**Yi**		**Han**		**Other**	
	**Number of clients**	**Proportion (%)**	**Number of clients**	**Proportion (%)**	**Number of clients**	**Proportion (%)**
Route of drug use	*χ*^*2*^ = 258.61, *P <* 0.0001					
Smoking or sniffing	3456	63.0	618	53.7	31	58.5
Pure injection	775	14.1	383	33.3	12	22.6
Injection mixed with other	1256	22.9	149	13.0	10	18.9
Sharing syringes	*χ*^*2*^ = 84.56, *P <* 0.0001					
Yes	939	17.1	317	27.6	22	41.5
No	4548	82.9	833	72.4	31	58.5

### Correlation of HIV-, HCV- and co-infection prevalence

There was a positive relationship between HIV and HCV EB adjusted prevalence among drug users across the townships (Pearson *r* = 0.263, *P* < 0.001), but the degree of relationship was not high. There was a strong positive relationship between HIV prevalence and HIV/HCV co-infection prevalence (Pearson *r* = 0.683, *P* < 0.001), and between HCV prevalence and HIV/HCV co-infection prevalence (Pearson *r* = 0.615, *P* < 0.001).

### Geographic cluster analysis of HIV infection

There were 396 townships with drug users tested for HIV infection and 222 townships without drug users tested for HIV infection. There was a significant Global geographic autocorrelation for HIV infection. The EB Rate Moran’s I was 0.3015 (*P* < 0.0001, 99999 permutations).

**Figure 2:**
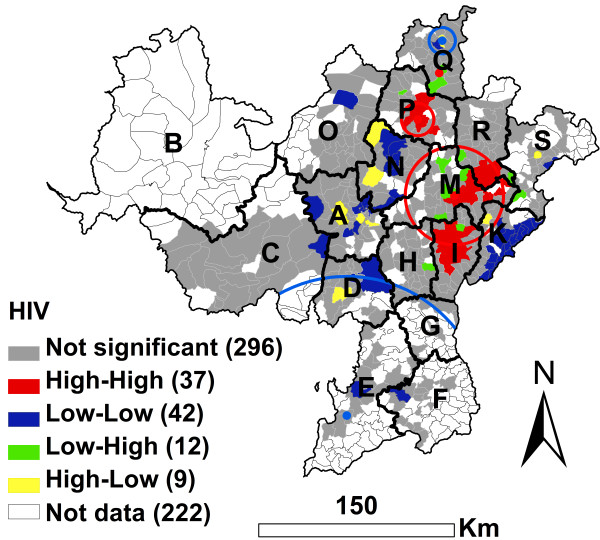
**EB adjusted LISA cluster map and geographic scan cluster locations of HIV infection among drug users in the national MMTP in Southwest China at the township level.** Red circles represent high risk clusters and blue circles represent low risk clusters.

For high prevalence of HIV infection, the results of the EB adjusted LISA (Local Indicators of Spatial Association) cluster analysis was similar to the results of the geographic scan cluster analysis (Figure 2). There were 37 High-High townships distributed in the I, M, R and P counties (Figure 2). The 37 High-High townships had 835cases (more than one-half of the total 1633 cases). The HIV infection prevalence of the drug users in these 37 townships was 41.8% (835/1998). The 9 High-Low townships had 45 cases and the infection prevalence of the drug users in the 9 townships was 23.9% (45/188). Outside the 37 High-High and 9 High-Low townships, there were only 753 cases with a 17.5% (753/4312) infection prevalence. 3 significant geographic high risk clusters of HIV infection were observed (*P* < 0.05) (Figure 2 and Table 3). These 3 clusters included 92 townships and 1122 cases (more than two-thirds of the total 1633 cases), with a 38.1% infection prevalence. Outside these 3 clusters, there were only 511 cases. The HIV infection prevalence of the drug users outside of these 3 clusters was 14.4%.

**Table 3:** Cluster information for HIV infection among MMTP clients using the geographic scan statistic in Southwest China

**Type of cluster**	**Number of towns**	**Cluster center (Latitude/longitude)**	**Radius (Km)**	**Population**	**Number of cases**	**Relative risk**	**Log likelihood ratio**	***P*** **value**
Most likely high risk cluster	80	27.9933 N, 102.8750 E	40.08	2714	1014	2.30	185.53	0.0001
Secondary high risk cluster	11	28.5176 N, 102.6218 E	11.46	201	89	1.81	18.00	0.0001
Secondary high risk cluster	1	28.8382 N, 102.7705 E	0.00	30	19	2.54	9.78	0.0289
Secondary low risk cluster	67	26.4770 N, 102.1328 E	114.25	394	23	0.22	53.92	0.0001
Secondary low risk cluster	7	29.0614 N, 102.7907 E	10.09	269	25	0.36	22.68	0.0001

For low prevalence of HIV infection, the results of local geographic autocorrelation analyses showed that there were 42 significant Low-Low townships which were not located in the I, M, R and P counties. These townships had only 63 cases, with a 7.5% (63/837) infection prevalence (Figure 2 and Table 3). The results of geographic scan statistics showed that there were 2 low risk clusters, which included 74 townships with a 7.2% (48/663) HIV infection prevalence. Most of the 74 townships were not Low-Low townships (Figure 2).

### Geographic cluster analysis of HCV infection

**Figure 3:**
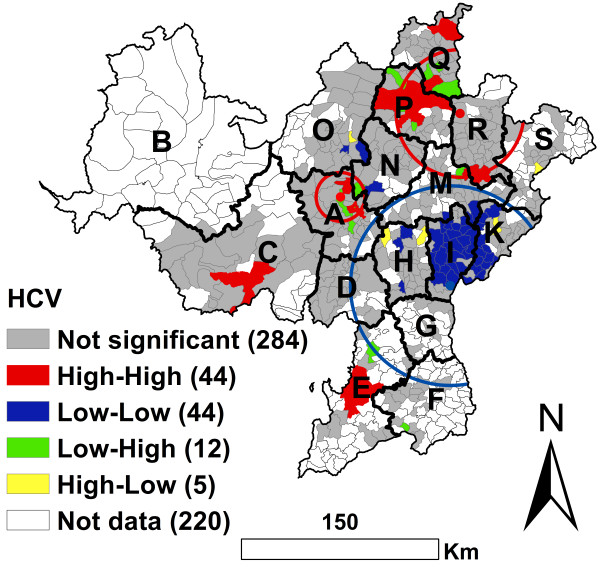
**EB adjusted LISA cluster map and geographic scan cluster location of HCV infection among drug users in the national methadone maintenance treatment program in Southwest China at the township level.** Red circles represent high risk clusters and blue circles represent low risk clusters.

There were 398 townships with drug users tested for HCV infection and 220 townships without drug users tested for HCV infection. There was a significant Global geographic autocorrelation for HCV infection. The EB Rate Moran’s I was 0.3449 (*P* < 0.0001, 99999 permutations). This result was similar to HIV infection, however, the local geographic distribution pattern of HCV infection was different from that of HIV infection (Figure 2 and Figure 3).

For high prevalence of HCV infection, the local geographic autocorrelation analysis showed that there were 44 significant High-High townships. These were mainly distributed in the A, C, E, P, Q and R counties (Figure 3). These High-High townships included 718 cases, with a 58.3% (718/1232) infection prevalence. Outside the High-High townships, there were 1017cases, with a 23.1% (1017/4395) infection prevalence. The geographic scan statistics identified 2 significant geographic high clusters of HCV infection, which included 1127 cases (approximately two-thirds of the total 1735) with a 49.9% (1127/2257) infection prevalence. Outside the high clusters there were only 608 cases, with an 18.0% (608/3370) infection prevalence (Figure 3 and Table 4).

**Table 4:** Cluster information for HCV infections among drug users, using the geographic scan statistic in Southwest China

**Type of cluster**	**Number of towns**	**Coordinates**	**Radius (Km)**	**Population**	**Number of cases**	**Relative risk**	**Log likelihood ratio**	***P*** **value**
Most likely low risk cluster	143	27.3696 N, 102.8758 E	78.64	2460	321	0.29	346.71	0.0001
Secondary high risk cluster	97	28.5478 N, 102.9428 E	55.73	1505	721	1.95	134.41	0.0001
Secondary high risk cluster	23	27.9769 N, 102.1250 E	19.14	752	406	1.98	101.19	0.0001

For low prevalence of HCV infection, the local geographic autocorrelation analyses identified 44 significant Low-Low townships which were mainly distributed in the I and K counties (Figure 3). These Low-Low townships had only 159 cases with a 9.7% (159/1631) infection prevalence. The geographic scan statistics identified 1 significant geographic low cluster which included most of the Low-Low townships (Figure 3). This low cluster included 143 townships with a 13.0% (321/2460) infection prevalence.

### Geographic cluster analysis of co-infection with HIV and HCV

**Figure 4:**
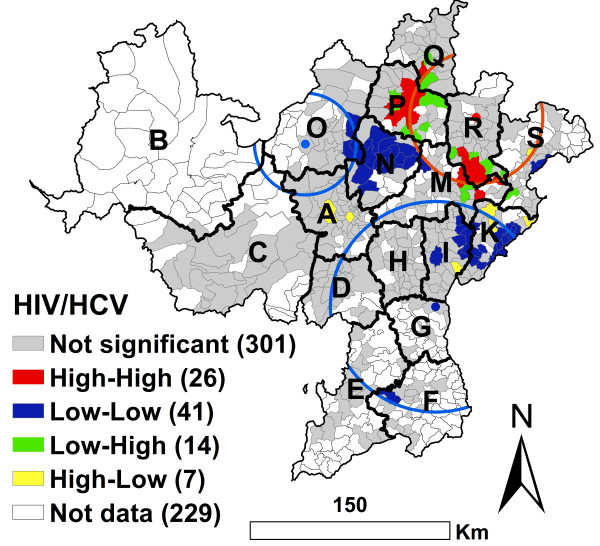
**EB adjusted LISA cluster map and geographic scan cluster locations of HIV/HCV co-infection among drug users in the national MMTP in Southwest China at the township level.** Red circles represent high risk clusters and blue circles represent low risk clusters.

There were 389 townships with drug users tested for both HIV and HCV infection. 229 townships without drug users tested for HIV or HCV infection. There was a significant Global geographic autocorrelation for HIV/HCV co-infection. The EB Rate Moran’s I was 0.3155 (*P* < 0.0001, 99999 permutations). This result was similar to both HIV and HCV infections (Figures 2, 3 and 4).

For high prevalence of HIV/HCV co-infection, the local geographic autocorrelation analysis identified 26 High-High townships. These were mainly distributed in the P and R counties. The 26 townships had 217 cases, with a 37.2% HIV/HCV co-infection prevalence. The geographic scan statistics analysis identified 1 significant high cluster, which included most of the High-High townships (Figure 4). This cluster had 323 cases (more than one-half of the total 590 cases), with a 27.8% (323/1163) HIV/HCV co-infection prevalence. Outside the cluster there were only 267 cases, a 6.3% (267/4263) HIV/HCV co-infection prevalence (Figure 4 and Table 5).

**Table 5:** Cluster information for HIV/HCV co-infection among MMTP clients, using the geographic scan statistic in Southwest China

**Type of cluster**	**Number of towns**	**Coordinates**	**Radius (Km)**	**Population**	**Number of cases**	**Relative risk**	**Log likelihood ratio**	***P*** **value**
Most likely high risk cluster	92	28.5087 N, 103.0829 E	55.71	1163	323	4.43	180.48	0.0001
Secondary low risk cluster	163	27.2293 N, 102.8077 E	88.14	2710	134	0.29	103.25	0.0001
Secondary low risk cluster	35	28.3288 N, 101.9280 E	46.04	262	7	0.24	13.08	0.0016

For low prevalence of HIV/HCV co-infection, the local geographic autocorrelation analysis identified 41 significant Low-Low townships. These were mainly distributed in the I, K, O and N counties (Figure 4). These Low-Low townships had only 12 cases, a 1.9% (12/623) HIV/HCV co-infection prevalence. The geographic scan statistics analysis identified 2 significant low clusters that included 198 townships (Figure 4 and Table 5). The 2 clusters had 141 cases, a 4.7% (141/2972) HIV/HCV co-infection prevalence. Outside the clusters, there were 449 cases, an 18.3% (449/2454) HIV/HCV co-infection prevalence.

## Discussion

The Yi Autonomous Prefecture is one of the largest illicit drug production and distribution centers of China. It is located along one of the main drug trafficking routes from the ‘Golden Triangle’ to northwest and central China [20]. The Prefecture has experienced an alarming increase in HIV infections since the first HIV case was identified among drug users in M county in 1995. This HIV epidemic has spread to all 16 counties and 1 city [20]. By the end of 2011, the cumulative number of individuals infected with HIV was over 25,000 [21]. We found the prevalence of HIV-, HCV- and co-infection were correlated across townships, similar to the previous reports [22-24]. The degree of relationship between HIV and HCV was not high. Our results from both the geographic autocorrelation analysis and the geographic scan statistical analysis showed that HIV-, HCV-, and co-infections in the Prefecture all exhibited significant geographic clustering at the township level. The geographic distribution patterns of these infections were clearly different. The areas of high HIV infection prevalence were mainly in the I, M, R and P counties where the proportion of Yi ethnic group was more than 50%. High HCV infection prevalence mainly occurred in the A, C, E, P, Q and R counties. Of these 6 counties, 3 (A, C, E) had a proportion of Yi population less than 50%. The high prevalence of co-infection with HIV and HCV mainly occurred in the P and R counties. These findings indicate that there might be some isolation of these communities, with more spread of infection within the communities than between them. The geographic heterogeneity of the two virus infections might be related to the geographic distribution of the ethnic groups involved. Our results revealed that HIV infection prevalence of the MMTP clients increased gradually, while HCV infection prevalence decreased gradually, as the proportion of Yi increased at the county level. This might be related to the special cultural traditions of the Yi ethnic group and the low proportion of the Yi injecting drug use or sharing syringes compared to the Han. This Yi ethnic minority still maintains traditional social norms and values including arranged marriage between members of the same social status and condoned causal sex. Hence, the transmission of these infections might be easier within some Yi communities than between them. Casual sex is also more frequent among Yi males than Han males, and unmarried Yi females have more frequent casual sexual behavior than married Yi females. Furthermore, condom use occurs infrequently among the Yi ethnic group [20,25]. More than 86% of the MMTP clients were less than 40 year old, so heterosexual transmission was more frequent among the Yi ethnic group than among the Han ethnic group. We also found that the proportion of Han both injecting drug and sharing syringes was higher than that of the Yi. Although HIV and HCV share routes of transmission, HCV is most efficiently transmitted via exposure to contaminated blood. For example, the transmission efficiency of HCV has been estimated to be some 10 times greater than that of HIV for needle-stick injuries, while the sexual and perinatal transmission of HCV is inefficient [1,9,26,27]. In A City with an 81.2% Han ethnic group, the HCV antibody prevalence among IDUs infected with HIV was 81.3% and the HIV/HCV prevalence ratio within the IDU population was 0.3. According to Vickerman et al. (2013) [28], the median estimated percentage of existing HIV infections due to sexual HIV transmission was only 38% in A city. In M County with a 97.1% Yi ethnic group, the HCV prevalence among IDUs infected with HIV was 57.5% and the HIV/HCV prevalence ratio within the IDU population was 1.3. Thus, the median estimated percentage of existing HIV infections due to sexual HIV transmission was 88% [28]. High HIV infection prevalence was more inclined to appear in some Yi cluster communities, while high HCV infection appeared in both Han cluster communities and Yi cluster communities.

Due to the shared routes of transmission, co-infection with HIV and HCV was very common in IDUs [1]. The HIV/AIDS epidemic in the Yi Prefecture was initially driven by transmission via injection drug use, and the majority of the early reported HIV cases occurred in young adults infected by injection drug use [20,25]. Among the 4 counties (I, M, R and P) with a high prevalence of HIV infection, the areas of high prevalence of co-infection with HIV and HCV were the P and R counties. County I had a low prevalence of co-infection with HIV and HCV. One reason for the substantial geographic heterogeneity in co-infection with HIV and HCV may be the difference in drug use behaviors between the two regions. The proportion of IDUs among the MMTP clients was 64.5% in R County and 52.1% in P County, but only 39.4% in I county. The other reason for this heterogeneity may be the difference in sexual HIV transmission between the two regions. The HCV antibody prevalence among IDUs infected with HIV was 86.7% in P County and 83.7 in R County. The HIV/HCV prevalence ratio within the IDU population was 0.64 in P County and 0.90 in R County. The median estimated percentage of existing HIV infections due to sexual HIV transmission was 55% in P County and 67% in R County [28]. However, in I County, the HCV prevalence among IDUs infected with HIV was 12.9% and the HIV/HCV prevalence ratio within the IDU population was 5.1. The median estimated percentage of existing HIV infections due to sexual HIV transmission was 88% [28]. These findings imply that intravenous drug use and heterosexual intercourse might be two important transmission routes in both P and R Counties, whereas in I county, heterosexual intercourse might be the dominate transmission route. The route of transmission in I county might be reflective of a change in the route of transmission from intravenous drug use to heterosexual sex. This needs further study. Analysis of surveillance data from the Yi Autonomous Prefecture showed that, over time, the percentage of intravenous drug use accounting for HIV transmission decreased, while transmission by heterosexual intercourse increased [29].

Our findings of substantial geographic heterogeneity of HIV and HCV infections at the township level imply that it is may be necessary to inform or invent site-specific intervention strategies to address the special cultural traditions of the Yi ethnic group in endemic areas and to better allocate currently limited resources to combat these two viruses. For example, P and R Counties have a high prevalence of co-infection with HIV and HCV. Because HIV/HCV co-infection in IDUs has become a rapidly emerging global public health problem [30,31], multiple approaches are imperative to prevent both HIV and HCV infections, identify new cases, and provide care. Local MMTP clinics should be strengthened and expanded using local multi-sector cooperation (e.g., public security and public heath sectors) to increase the coverage and retention of MMTP and improve accessibility of services [11]. Thus, more drug users will be engaged in the MMTP clinics and receive more effective interventions, including uninterrupted drug substitution treatments, and both HIV and HCV testing. Effective prevention and treatment for both infections will be available and provide an ability to halt the tide of increasing morbidity and mortality due to HCV/HIV-related disease.

There are some limitations to our study. First, only a part of all the drug users enrolled in the MMTP clinics. It is difficult to confirm whether MMTP participants are a truly representative sample of a population of community-dwelling drug users. However, it has been estimated that the national HIV prevalence among MMTP clients is not significantly different from that among non-MMTP drug users reported by national sentinel surveillance during 2004–2009 [12]. Second, HIV and HCV are transmissible infections, and they often share similar transmission routes. Therefore, the clustering found with the spatial methods might be related to the clustering of risk behaviors, to natural geographic boundaries between human populations, or to both. However, due to the limitations of these analytic approaches (e.g., they did not consider HIV, HCV and co-infections as communicable diseases, and are limited in the ability to identify which reason may contribute to the clustering), we cannot analyze unambiguously what resulted in the clustering. In addition, the information regarding the use of condoms and sexual behaviors is not available and individual risk factors were not considered in the framework of our geographic analyses. These limit the ability to further analyze why the geographic distribution of these infections in our study field was heterogeneous. Some studies show that casual sexual behaviors are frequent, and condoms were infrequently used among the Yi ethnic group compared to the Han ethnic group [20,25].

## Conclusions

This was the first study to analyze the geographic distribution patterns of HIV-, HCV, and co-infections among drug users in a national MMTP in a Yi Autonomous Prefecture, Southwest of China. These infections exhibited substantial geographic heterogeneity at the township level. Their geographic distribution patterns were also obviously different. These findings imply that it is may be necessary to inform or invent specific intervention strategies, particularly regarding the Yi ethnic group. Currently limited resources will need to be reallocated according to these findings.

## Abbreviations

HIV: Human immunodeficiency virus type 1; HCV: Hepatitis C virus; IDUs: Intravenous drug users; MMTP: Methadone maintenance treatment program; EB: Empirical bayes; GIS: Geographical information systems.

## Competing interests

The authors declare that they have no competing interests.

## Authors’ contributions

YBZ, QXW, YHG, SJN, LN, AHY, QL, and XXS contributed to collection and management of data. YBZ, SL, and QWJ contributed to the data analysis and wrote the manuscript. All authors contributed to the interpretation of data, read and approved the final manuscript.

## Pre-publication history

The pre-publication history for this paper can be accessed here:

http://www.biomedcentral.com/1471-2334/14/134/prepub
